# Spheroids of FAP-Positive Cell Lines as a Model for Screening Drugs That Affect FAP Expression

**DOI:** 10.3390/biomedicines11072017

**Published:** 2023-07-18

**Authors:** Victor V. Pleshkan, Marina V. Zinovyeva, Dina V. Antonova, Irina V. Alekseenko

**Affiliations:** 1 Gene Immunooncotherapy Group, Shemyakin-Ovchinnikov Institute of Bioorganic Chemistry of the Russian Academy of Sciences, 117997 Moscow, Russia; mzinov@mail.ru (M.V.Z.); tyulkina.dina@mail.ru (D.V.A.); irina.alekseenko@mail.ru (I.V.A.); 2 National Research Center “Kurchatov Institute”, 123182 Moscow, Russia; 3 Laboratory of Epigenetics, Institute of Oncogynecology and Mammology, National Medical Research Center for Obstetrics, Gynecology and Perinatology Named after Academician V.I. Kulakov, Ministry of Healthcare of the Russian Federation, 117198 Moscow, Russia

**Keywords:** fibroblast activation protein, cancer-associated fibroblasts, 3D spheroids

## Abstract

Fibroblast activation protein has a unique expression profile that manifests mainly in wounds and tumors, which anticipates it as an encouraging and selective target for anticancer therapy. However, research of the therapeutic potential of FAP is limited both by legal restraints when working in vivo and by the difficulty of obtaining standardized primary cultures of FAP-positive cancer-associated fibroblasts due to their high heterogeneity. We found that 3D spheroids of FAP-positive cell lines could serve as robust and convenient models of FAP expression, in contrast to monolayers. By exposing such spheroids to various factors and compounds, it is possible to study changes in FAP expression, which are easily detected by confocal microscopy. FAP expression increases under the influence of the TGFβ, does not depend on pH, and decreases during hypoxia and starvation. We believe that the proposed model could be used to organize large-scale high-throughput screening of drugs that target FAP expression.

## 1. Introduction

Over the last decade, tumor–stroma crosstalk has been considered as a promising therapeutic target [1]. Such crosstalk is generated by interactions between malignant and nonmalignant cells, including immune system cells, the tumor vasculature, and a heterogeneous population of fibroblast-like cells called cancer-associated fibroblasts (CAFs), which together form the tumor microenvironment (TME) [2]. Meanwhile, stroma cells regulate cancer cell biology and the TME through intercellular contacts [3]. This regulation leads to the release of numerous regulatory factors and remodeling of the extracellular matrix (ECM), which contributes to cancer progression [4,5]. Changes in the ECM are the result of an organized cross-interaction between cancer and stroma cells. In this process, stroma cells are activated by a number of mediators released by cancer cells, including paracrine factors, cytokines, etc. In turn, activated stroma cells secrete numerous regulatory factors that promote tumor development [6,7] and affect ECM [8]. Due to the mutual support between cancer cells and the stroma microenvironment, affecting any one of them is unlikely to break the vicious circle [9]. Thus, the concept of disrupting pro-tumor interactions within a tumor to make cancer cells vulnerable to the immune system and anticancer drugs is currently the subject of extensive discussion [10,11]. 

Cancer-associated fibroblasts are the most prominent elements of tumor stroma [12,13]. They provide reciprocal interaction between the cancer cells and cells of the TME, both through direct interactions and paracrine signaling [14]. Among these interactions, the fibroblast activation protein (FAP) plays a crucial role. FAP was originally identified as an inducible antigen expressed on reactive stroma and represents one of the main markers of corrupted by tumor fibroblasts—CAFs [15,16]. Its unique expression predominantly in tumors rather than normal adult tissues makes it a selective target [17]. 

FAP belongs to class II transmembrane serine proteases and possesses two types of enzymatic activity: dipeptidyl peptidase and endopeptidase [18]. The truncated form of FAP is a soluble protein that can be located outside and inside cells. [19,20]. It is assumed that FAP exhibits enzymatic activity only as a 170-kda homodimer with two N-glycosylated subunits [20,21,22]. FAP remodels the extracellular matrix (ECM) through its own enzymatic activity, thereby affecting the invasiveness and many other properties of cancer cells [23,24,25]. Another important signature of FAP is the provision of certain properties of the microenvironment, which depend little on its enzymatic activity. Thus, research conducted on a mouse model of breast cancer showed that the introduction of a catalytically inactive mutant form of FAP into cancer cells does not lead to suppression of inoculated tumor growth, although it is necessary for the proteolytic degradation of ECM [26]. Moreover, it has been shown that cell growth and motility in breast cancer do not depend on the catalytic activity of FAP, but can be regulated by other signaling pathways [27]. There is ample evidence that FAP contributes to TME immunosuppression, thereby causing rapid tumor growth and treatment resistance [28,29]. For example, FAP-positive pancreatic ductal adenocarcinoma (PDA) cells have been shown to overexpress the CXCL12 chemokine, resulting in disruption of the antitumor effects of α-CTLA-4 and α-PD-L1 immunotherapy, the effect of which was restored by administration of AMD3100, a CXCL12 receptor chemokine (C-X-C motif) receptor 4 inhibitor [30]. 

Therefore, both due to enzymatic activity and independently of this activity, FAP can exhibit protumor activity, including migration, invasion, proliferation of stroma fibroblasts, cancer and endothelial cells, causing stromal degradation, epithelial-mesenchymal transition, tumor angiogenesis, and immunosuppression [31]. The transmembrane form of FAP is of greater therapeutic interest—such localization allows it to effectively perform enzymatic functions for ECM remodeling and is of interest for theranostics [32,33]. Thus, the search for factors affecting FAP expression is of great importance for the possibility of its use in anticancer therapy. However, FAP-positive CAFs are typically a fraction of cells that are difficult to standardize for in vitro culturing (primary cultures may be a set of heterogeneous cells), which can make large-scale screening of potential therapeutics tricky. Previously, we have identified several FAP-positive cell lines—RMS (SJCRH30), OSA (SJSA-1), and NGP-127, which can serve as convenient and reproducible models for studying the regulation of FAP expression [34]. In these cell lines, the level of FAP expression was comparable to stroma samples from primary cultures of lung and pancreatic cancer both at the translational and protein levels. 

In this work, we have exploited and evaluated an approach to use 3D models of FAP-positive cell lines as a model for screening drugs that affect FAP expression. The proposed approach could allow us to quickly conduct a large-scale preparation search without obtaining CAFs or use of animal models.

## 2. Materials and Methods

### 2.1. Cell Lines

Cell lines of rhabdomyosarcoma SJCRH30/RMS 13 (ATCC, CRL-2061) and osteosarcoma SJSA-1 (ATCC, CRL-2098) were obtained from the American Type Culture Collection (ATCC, Manassas, VA, USA). Neuroblastoma NGP-127 cell lines were kindly provided by Paul S. Meltzer. Cells were cultured in the DMEM/F12 (1:1) medium supplemented with 100 units/mL, penicillin, 100 μg/mL streptomycin, and 0.25 μg/mL amphotericin and 10% fetal bovine serum and were maintained in a humidified atmosphere at 5% CO_2_ and 37 °C. The media and supplements were purchased from Gibco/Thermo Fisher Scientific (Waltham, MA, USA).

### 2.2. Obtaining of Spheroids

For 3D culture analysis, spheroids were formed in 24-well plates precoated with polyHEMA (Poly(2-hydroxyethyl methacrylate)) (Sigma-Aldrich, St. Louis, MO, USA). To do this, 150–200 µL of polyHEMA polymer in 96% ethyl alcohol at a concentration of 5 mg/mL was applied to each well of a 24-well plate, after which the plate was dried under a lamp until the alcohol solution completely evaporated. Next, 500 000 cells were seeded in the prepared well, and the cells were cultured as spheroids for 72 h before being used for any of the assays presented in this study.

### 2.3. Immunofluorescent Staining

Next, 3D immunofluorescent staining of FAP in cells of various lines was performed by using as primary antibodies FAP Antibody (F11-24): sc-65398 (Santa Cruz Biotechnology, Dallas, TX, USA) and as secondary antibodies conjugated with fluorescent dye–Goat anti-Mouse IgG Alexa488 (Thermo Fisher Scientific, Waltham, MA, USA). The membrane was stained with WGA Alexa Fluor 594 (Thermo Fisher Scientific, Waltham, MA, USA). Hoechst 33342 (Sigma-Aldrich, St. Louis, MO, USA) was used to visualize nuclei. Slides were analyzed using Eclipse TE2000 confocal microscope (Nikon, Minato-ku, Tokyo, Japan).

#### 2.3.1. Immunofluorescent Staining of Spheroids

After culturing the spheroids for 72 h in a 24-well plate, they were transferred to 200 μL of culture medium in a 96-well plate, and immunofluorescent staining was performed. Primary antibodies sc-65398 were added to the samples at a concentration of 0.5 μg/mL and incubated at 37 °C for 1 h. Then, 30 min before the expiration of one hour of incubation with primary antibodies, membrane dye WGA Alexa Fluor 594 was added to the samples at a concentration of 0.5 μg/mL and incubation continued at 37 °C. Next, the medium was removed and the cells were fixed in 2% PFA for 20 min at 37 °C. Then, PFA was removed and the cells were resuspended in 100 μL PBS, then secondary antibodies were added to the cells at concentration of 0.5 μg/mL and incubated at 4 °C overnight. Next morning, the cells were placed on cover slips, incubated at RT for 10–15 min, and then the spheroids were glued to slides using the ProLong™ Gold Antifade Mountant (Thermo Fisher Scientific, Waltham, MA, USA). As control samples, untreated spheroids and spheroids treated only with secondary antibodies were used.

#### 2.3.2. Immunofluorescence Staining of Monolayer Cultures

Staining was performed on cover slips, on which 100,000 cells were preliminarily seeded. Then, 200 μL of growth medium containing primary antibodies at concentration of 0.5 μg/mL and WGA Alexa Fluor 594 at concentration of 0.5 μg/mL were added to each slide and incubated at 37 °C for 1 h. Next, cells were fixed in 2% PFA and incubated for 15 min at 37 °C. Cells were washed with PBS and secondary antibodies were added at a concentration of 0.5 µg/mL and incubated for 15 min at 37 °C. Cover slips with cells were slightly dried and glued to the slides with ProGold™ Gold Antifade Mountant. Untreated spheroids and spheroids treated only with secondary antibodies were used as control samples.

### 2.4. Changing Cell Culturing Conditions

To evaluate the effect of the transforming growth factor-beta (TGFβ) on FAP expression, spheroids were cultured with the addition of the TGFβ1 at concentration of 10 ng/mL, while the FCS content in the growth medium was reduced to 0.5% to neutralize the effect of serum. In this case, the medium was changed every 24 h. As a control, spheroids cultured in DMEM/F12 (1:1) medium containing 0.5% FCS without the addition of TGFβ1 were used. Spheroids cultured in DMEM/F12 (1:1) medium with 0.5% FCS were used as a sample cultured under growth factor depletion conditions when compared to spheroids cultured in 10% FCS. 

To evaluate the influence of the pH, the cells were cultured in DMEM/F12 (1:1) medium with 0.5% FCS and acidity of was adjusted to pH = 6.0, while the acidity of the routinely used medium was approximately pH ≈ 7.0. The medium was changed every 24 h. 

To simulate hypoxia inherent for tumors, spheroids were cultured in DMEM/F12 (1:1) medium with 10% FCS supplemented with cobalt chloride (CoCl2) at concentration of 150 μM. Immunofluorescent staining was performed 24 h after adding CoCl_2_ to spheroids.

### 2.5. Real-Time PCR

The transcription level of the *FAP* gene in spheroids was evaluated by real-time PCR. Simultaneously with immunofluorescent staining, aliquots of the spheroids were collected for subsequent isolation of total mRNA. To isolate, total RNA cells were trypsinized and washed twice with PBS. Total RNA was isolated using an RNeasy Mini Kit (Qiagen, Venlo, The Netherlands) followed by treatment with DNAse RQ1 (Promega, Madison, WI, USA) according to the manufacturer’s protocol. The quality of RNA was analyzed by electrophoresis in a 1% agarose gel containing ethidium bromide. The amount of RNA was determined with a NanoDrop 2000 spectrophotometer (Thermo Fisher Scientific, Waltham, MA, USA) at the absorption wavelength of 260 nm. 

The transcription level of the genes under the study was evaluated by qPCR using a qPCRmix-HS SYBR reaction mixture (Evrogen, Moscow, Russia). The first cDNA strands were synthesized using hexanucleotide primers and Mint reverse transcriptase (Evrogen, Moscow, Russia) according to the manufacturer’s protocol. For real-time PCR, primers FAP-for-S_E15 (5’-CAGCAAGTTTCAGCGACTAC-3’) and FAP-rev-S_E19 (5’-CAGCAAATACAGACCTTACAC-3’) were used. Transcription level of *FAP* gene was normalized relative to the geometric mean of the transcription level of the 18S RNA, the *GPI*, and the *EEF1A1* genes as it was described earlier [35].

#### Statistical Analysis

Statistical processing of the data was performed using Microsoft Excel 2013, LinRegPCR (Version 2012.2), and LC480Conversion (Version 2.0). The data were deemed significant at *p* < 0.05.

## 3. Results

### 3.1. Rationale for the Method Used

The failure of therapy directed at targets within cancer cells has shifted the attention of researchers towards disrupting the interaction of cancer cells with the tumor microenvironment [36]. One of these interactions are direct binary contacts between ligands and receptors exposed on the surface of cancer and stroma cells. Effective cancer treatment should not target individual components of hypercomplex intracellular interactomes (molecular targeting), but rather disrupt intercellular interactions between cancer and stroma cells, thereby destroying the tumor as a whole [11]. From this point of view, transmembrane FAP is an extremely intriguing target. Therefore, the search for relevant models and methods to study the influence of various factors on FAP expression is a critical mission for the possibility of using this challenging target for therapeutic applications. To determine the cellular localization of FAP, different approaches are used. The most optimal methods seem to be flow cytometry, confocal microscopy, and NHS-biotinylation [22,37,38]. Since FAP localization could be of certain interest from a functional and therapeutic point of view, we decided to use confocal microscopy as a base for our research. This method gave us the most reliable and informative data.

### 3.2. Choice of a Model for Research

We have previously shown that standard cultured cell lines can be a source of FAP [34]. To be used as a model, cells should express a transmembrane form of FAP. Therefore, we need to determine FAP localization in the FAP-positive cell lines using immunofluorescent staining and confocal microscopy. Monolayer cultured cells and spheroids were used for this purpose. Spheroids have a higher density of cellular contacts, which brings them closer to tissues, with their inherent intercellular interactions. Thus, we expected to see a higher FAP expression there compared to a monolayer. 

In order to perform such experiments, after culturing under standard conditions, cells of FAP-positive cell lines were seeded on coverslips to obtain a monolayer or in 24-well plates coated with pHEMA to form spheroids. To determine the level of FAP protein expression, we performed immunofluorescent staining of FAP protein in the monolayer and spheroids of FAP-positive cell lines using primary antibodies for FAP and secondary antibodies conjugated with Alexa480 fluorescent dye. Simultaneously, the cell membrane was stained with the Alexa Fluor 594 WGA dye. 

For all series of experiments with immunofluorescent staining, two sets of samples were prepared: experimental samples—FAP-positive cell lines, RMS (SJCRH30), OSA (SJSA-1), or NGP-127 cells, which can serve as a convenient and reproducible model for studying the regulation FAP expression. As control, the FAP-negative HEK293 cell line was used. All samples in each experiment were processed simultaneously in the same way. All samples were stained as follows: (1) immunofluorescent staining of the FAP protein using primary antibodies to FAP and secondary antibodies conjugated to the fluorescent dye Alexa480; (2) cell membrane staining with Alexa Fluor 594 WGA dye; (3) Hoechst 33342 nuclei staining. In confocal microscopy, sample positioning was performed by detecting nuclei stained with Hoechst. More accurate positioning was performed by stained cell membranes. Taking images in the red (membrane) and green (FAP) channels was performed at the same positioning, amplification settings, and fluorescent signal accumulation, which made it possible to obtain images of same microscopic section. In all cases, the experiment began with verification of the FAP-positive samples. After confirming the presence of green luminescence, the absence of luminescence of FAP-negative samples was determined in the same channel. Thus, we confirmed that the green color corresponds precisely to FAP expression. Further, in the text, all descriptions of negative control manipulations are omitted. An example of the use of all stainings and controls is given in Supplementary Figure S1.

**Figure 1:**
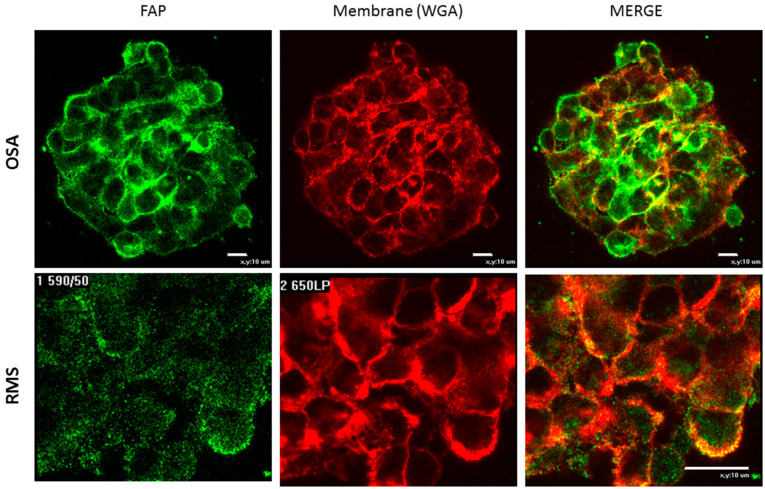
Representative confocal microscopy images of FAP-positive cell line spheroids. FAP expression (green) is co-localized with cell membranes (red). The name of the cell line is indicated on the left side. The RMS cell line is shown at a higher magnification than the OSA line. The scale (white horizontal line) is indicated in the lower right corner and corresponds to 10 µm.

**Figure 2:**
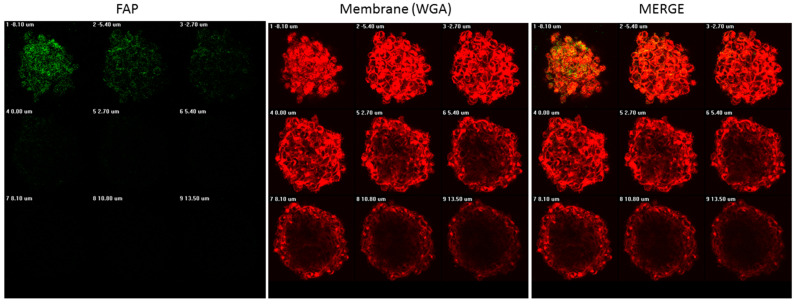
Z-stack for spheroid of the RMS cell line. In the upper left corner, there is an image corresponding to the most extreme position of the start of scanning the spheroid. From left to right and further down, the scan layer shifts. It can be seen that the highest concentration of the FAP protein is located exactly on the surface of the spheroid.

It was shown that in the monolayer culture the level of the FAP fluorescent signal was extremely low and comparable to the level of autofluorescence, while in 3D cultures, FAP expression was detected at a high level. The overlap of signals in the red channel (membrane) and green (FAP) indicated that FAP expression is predominantly co-localized with the cell membrane (see Figure 1). Thus, only the use of spheroids gave an adequate image, and further on we used spheroids. Interestingly, the localization of the FAP protein in the spheroid is uneven and is more characteristic of its surface (Figure 2). As can be seen, the strongest signal from the FAP is located at the top of the spheroid, which is represented by the surface with the largest area. However, as the layer goes deeper, the signal becomes weak and blurry. This fluorescent signal loss throughout the z-depth of spheroids is known as a reproducible, exponential decay function [39]. At the same time, signal loss from FAP occurs much faster than from membrane staining, which rather indicates its more specific surface distribution.

### 3.3. Evaluation of Chosen Model

#### 3.3.1. TGFβ Activates FAP Expression in 3D Spheroids of FAP-Positive Cell Lines

To confirm the relevance of this model, it is necessary to demonstrate that the regulation of FAP expression in FAP-positive cell lines is similar to its regulation in CAFs. According to numerous works, the transforming growth factor-beta (TGFβ) is the main activator of FAP [40,41,42]. For example, TGFβ signaling upregulates the expression of CAF markers such as α-SMA (alpha-smooth muscle actin) and FAP, promoting the activation of CAFs [43]. TGFβ-activated CAFs promote tumor invasion, pulmonary metastasis, and EMT, acting particularly through overexpression of FAP [44].

**Figure 3:**
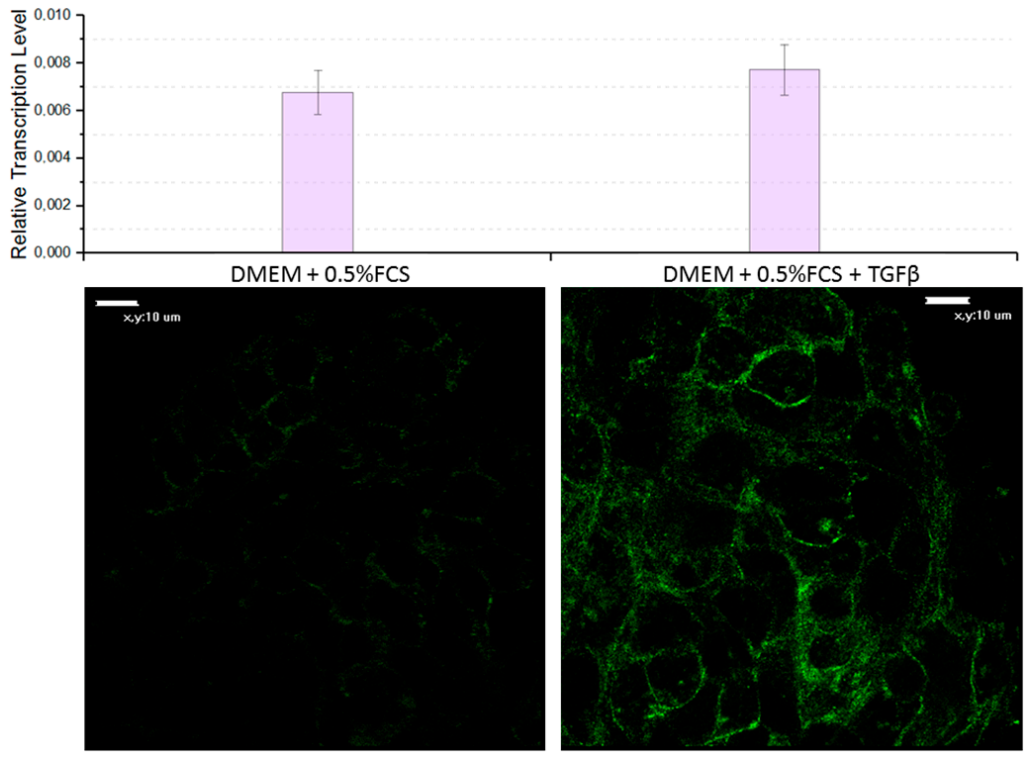
FAP expression is increased by adding TGFβ to OSA spheroids. Upper side—relative transcription levels of the *FAP* gene when a spheroid is cultured in DMEM/F12 containing 0.5% FCS with or without supplementation of the TGFβ. The transcription level was calculated relative to the geometric mean level of transcription of the 18S, *GPI*, *EEF1A1* genes. Lower side—representative confocal microscopy images of a spheroid grown in a DMEM/F12 medium containing 0.5% FCS with or without supplementation of the TGFβ.

To perform this verification, spheroids were treated with TGFβ under conditions of 0.5% fetal bovine serum depleted medium. This was carried out in order to neutralize the influence of the TGFβ present in the serum, as well as to prevent the binding of TGFβ added to the medium with serum proteins. It was shown that during the cultivation of spheroids of FAP-positive cells in presence of the TGFβ, an increase in FAP expression was observed (see Figure 3), both at the protein and mRNA levels, in contrast to cells that do not express FAP. The results are consistent with an increase in FAP expression in CAFs responding to the TGFβ [45].

#### 3.3.2. Positive TGFβ-Feedback Loop in Spheroids

Since many tumor cells can be a source of the TGFβ [46,47], we checked the expression level of the *TGFβ* gene in the cells we used. It was found that all three studied cell lines expressing FAP are positive for *TGFβ* transcription (data not shown). 

Previously, it was shown that the conditioned medium of cancer cells can stimulate fibroblasts due to the accumulation of cell-synthesized TGFβ [41]. During cell culturing the products of cellular metabolism accumulate, including secreted proteins, e.g., TGFβ. Therefore, it can be assumed that the amount of TGFβ will increase in the conditioned medium if it is not changed. Thereby, FAP expression will be more strongly activated. To test our hypothesis, we cultivated spheroids of the NGP-127, RMS 13, and OSA cell lines with and without replacement of the growth medium. 

**Figure 4:**
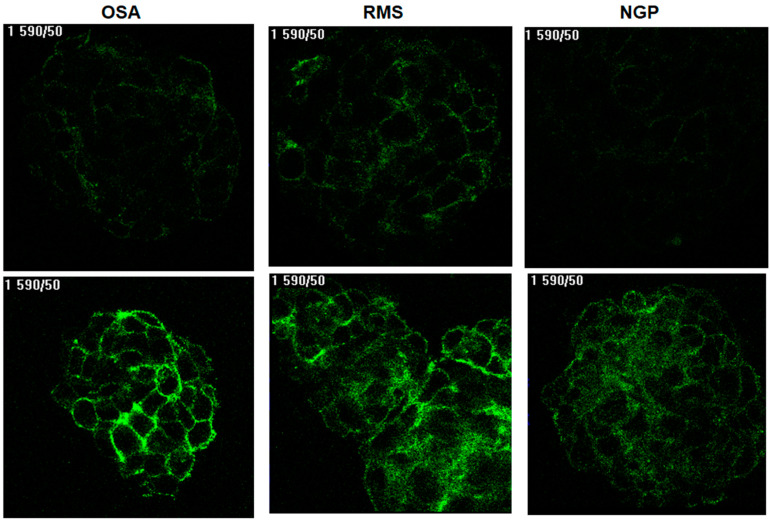
An increase in the level of FAP expression in spheroids of cell lines OSA, RMS 13, NGP-127 (cell line names are indicated at the top of the figure) during cultivation without changing the medium. Cell spheroids were cultured in DMEM/F12 medium for 72 h with a single medium change (upper row of the images) or no medium change (lower row of the images).

It was shown that during spheroids culturing without changing the medium (in conditioned medium) for 72 h, the expression of the FAP protein is increased compared to spheroids culturing with changing the medium (Figure 4). As one can see, the levels of FAP expression are increased in spheroids from different FAP-positive cell lines. Thus, the spheroids respond in the same way, but with various extents.

Therefore, prolonged incubation of cells in a conditioned medium leads to an increase in FAP expression on the cell surface. The accumulation of TGFβ in the medium can stimulate the expression of the FAP protein under conditions of a rare change in the nutrient medium. That is, these cells may have a positive TGFβ-FAP autoregulatory loop, which was previously shown in a broad spectrum of cell types present in the glioblastoma microenvironment [42].

#### 3.3.3. Impact of Various Factors on FAP Expression in 3D Spheroids

The effect of different culturing conditions on FAP expression was tested on OSA spheroids. Conditions were modeled that may correspond to the pathophysiological niche of the tumor were hypoxia, low pH, changes in the concentration of nutrients, and growth factors. It is known that when cells are cultured, acidification of the medium occurs, and the tumor itself is characterized by lower pH values compared to normal tissues [48,49]. This led us to hypothesize a possible effect of pH on FAP expression. To test this hypothesis, a medium with an increased acidity (up to pH 6.0) was used. The cells were cultured in media with pH 6.0 and pH 7.0 (usual normal medium). In both cases, the medium was changed at the same time. However, there was no significant difference in FAP protein expression (data not shown). 

**Figure 5:**
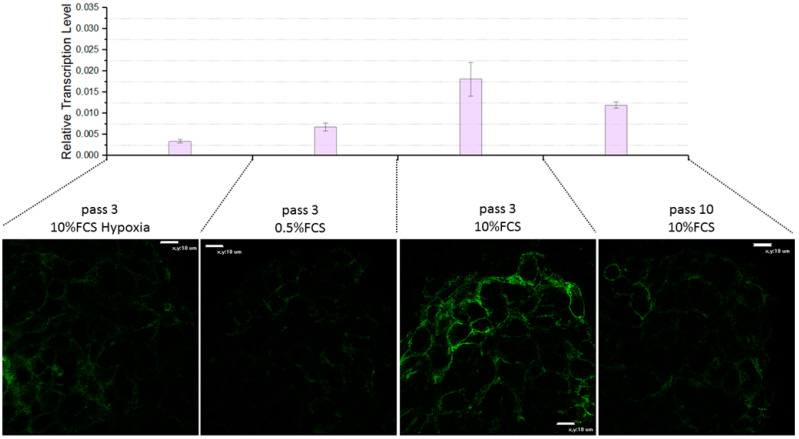
FAP expression upon incubation of OSA spheroids under different conditions. Conditions for cultivation and passage of cells used for spheroid formation are indicated above the confocal image of the spheroids. The transcription level of *FAP* gene was calculated relative to the geometric mean level of transcription of the 18S, *GPI*, *EEF1A1* genes.

Another feature of the tumor physiology is hypoxia, associated with the peculiarities of the blood supply of tumors due to their rapid growth [50,51]. We used a model of hypoxia initiated by cobalt chloride. FAP expression was shown to be inhibited under conditions of cobalt hypoxia (see Figure 5; 10% FCS hypoxia).

The rapid proliferation of cancer cells is due to the high amount of growth factors that affect the tumor and its environment. During cell culturing, the main source of such factors is FBS. By reducing its amount, it is possible to simulate the effect of reducing the impact of growth factors. To simulate growth factor reduction, we reduced FBS from 10% to 0.5% with the same amount of glutamine. Since it is known that L-glutamine serves as an auxiliary energy source, especially when cells are rapidly dividing [52]. It has been shown that FAP expression is inhibited in a medium depleted in FBS (see Figure 5; 0.5% FCS). 

Thus, it has been shown that a decrease in FAP expression occurs in response to conditions of cobalt hypoxia and depletion in growth factors or nutrients, while the pH of the culturing medium does not affect expression. The transcription level of the *FAP* gene under conditions of depleted nutrient medium and cobalt hypoxia is strongly reduced compared to cells cultured under normal conditions (see Figure 5; 10% FCS).

### 3.4. Limitations of the Proposed Model System

During the repetition of experiments by passaging of cell lines used to obtain spheroids, a decrease in the intensity of the FAP protein fluorescence signal was observed, while the level of the nuclear and membrane staining signal did not change its intensity. In this case, the experimental conditions were the same. To identify the reasons for the fade in the FAP fluorescence signal, we studied the effect of successive passivation of FAP+ cells under conventional conditions (standard plastic for cell cultures). Data have previously been published suggesting that in the absence of a substrate (e.g., collagen-coated flasks), FAP expression may be inhibited [53]. 

To determine the change in the expression level of the FAP protein during long-term culturing, immunofluorescence staining of the FAP protein in spheroids of FAP-positive cell lines was performed after 10 passages of cell line culturing. It was found that the level of FAP expression decreases with increasing cell passages: FAP fluorescence is practically absent after the 10th passage (see Figure 5; pass 10, 10% FCS). The transcription level of the *FAP* gene also decreases with increasing cell passage (see Figure 5). This peculiarity of FAP expression should be taken into account when conducting experiments and comparing the results of different studies.

## 4. Discussion

The unique expression of FAP predominantly in CAFs makes it an attractive therapeutic target, which has been the subject of many studies. At the same time, studies of the expression and transcriptional regulation of FAP show contradictory results [54]. The exhibition of diverse FAP activities, often not associated with enzymatic activity; the presence of two forms (soluble and membrane-bounded); and the complexity of expression regulation raise difficulties in studying the factors affecting its expression. The specialness of FAP expression in CAFs is rather an obstacle for research. Thus, it is almost impossible to obtain a standardized FAP-positive material due to the high heterogeneity in the expression levels of *FAP* and other marker genes in CAFs [55,56,57]. In the present work, we propose a simple model for testing the impact of various factors on *FAP* gene expression. 

Previous studies implemented by us and other authors have shown that some cell lines express FAP at a level comparable to those in CAFs. It can be assumed that the mechanism of expression regulation in such cell lines is similar to the regulation of FAP expression in CAFs. This can be confirmed by the influence of the TGFβ on the increase in the level of FAP expression on the surface of spheroids, which was shown in this work. Our results demonstrate that the main regulation of the *FAP* gene in FAP-positive lines depends on and is regulated by the TGFβ, which is consistent with what researchers have observed for CAFs [57]. Apparently, there is an autoregulatory TGFβ loop in FAP-positive cell lines, which to some extent can mimic complex intratumoral interactions, when stroma–tumor paracrine signals serve to feed each other by repeatedly reflecting and multiplying. Possibly, it may reflect cancer progression, when tissue-resident normal fibroblasts are gradually transformed into CAFs, and the activation of autocrine signaling pathways mediated by TGFβ and SDF1 (stromal cell-derived factor-1) is initiated, which promotes CAF formation via self-stimulation and cross-communication [58]. FAP expression is independent of pH and probably cannot be used for local activation of FAP of targeted drugs. At the same time, depletion in growth factors associated with a decrease in FAP expression provides wide opportunities for identifying individual factors that are important for FAP expression, but are not required for adult tissues, which can be used for tumor-specific ligand-receptor targeting. The increase in protein expression observed by us was also confirmed by an increase in the transcription of the *FAP* gene. This suggests that the observed effect is mainly regulated at the transcriptional level (at least partially), which was previously described for the *FAP* gene [59]. 

Thus, our model mimics the properties of CAF in the tumor microenvironment and may serve as a simple model to allow for a broad screening of agents potentially affecting FAP expression. Although these studies will not conclusively answer the mechanism of action of the suppression of FAP expression and its consequences for the body, the introduction of such a stage for screening potential drugs before conducting preclinical studies can significantly expand the base of tested drugs. To conduct such research, several conditions should be fulfilled:

- Find (in your lab) any FAP-positive cell line suitable for routine maintenance;
- Verify that the main FAP-activation signaling pathway-TGFβ works or try to use another pathway you know (or need to test). This should be control of response;
- Use spheroids of the FAP-positive cell line instead of monolayer cultures;
- Use of earlier passages of cell lines to obtain spheroids is preferable.

In this way, a number of compounds of interest can be tested. Concurrently, the intensity of FAP fluorescence can serve to assess the quantitative effect of exposure to various compounds. It is worth noting that activation of CAFs by the TGFβ are characteristic for myofibroblastic fractions of tumor CAFs, which are known as pro-tumor CAFs [60]. Meanwhile, other CAF fractions, such as inflammatory CAF (iCAFs) or “antigen presenting CAF” (apCAF), might have their own regulatory pathways [61]. We speculate that the use of specifically responding CAFs as “control of response” in such analysis might contribute to finding selective anti-tumor drugs targeting TME. However, further research is required to confirm this assumption.

In this study, we proposed a model that allows, in a simple system, to reliably observe changes in FAP expression at the protein level in a renewable, easily reproducible material. Our approach does not require addressing the special regulatory issues inherent for in vivo models and studies using human biomaterial. In general, the use of our method allows for a broad screening that answers the question of whether this compound/condition affects FAP expression and how. For further research, it will be necessary to use animal models to assess the overall effect of the compounds/conditions found on the organism as a whole. 

We believe that our approach can be used to screen a large number of candidate molecules, which are then verified in other systems that do not allow such large-scale screening. This will speed up and reduce the cost of finding FAP-based drugs.

## Data Availability

The data presented here are available throughout the Article and Supplementary Materials.

## Supplementary Materials

The following supporting information can be downloaded at: https://www.mdpi.com/article/10.3390/biomedicines11072017/s1, Figure S1: Cell line staining, 2D and 3D.
