# Supplementary material for: Spheroids of FAP-Positive Cell Lines as a Model for Screening Drugs That Affect FAP Expression
Source: Biomedicines. 2023 Jul 18;11(7):2017. doi: 10.3390/biomedicines11072017 (PMC10377737; doi:10.3390/biomedicines11072017)

HEK293 FAP-negative cell line

The difference between 2D and 3D cultures of the HEK293 cell line is indicated.

1ry + 2ry Antibodies – image taken when primary (FAP antibody) and secondary antibody conjugated with fluorescent dye – Goat anti-Mouse IgG Alexa488 were used.

Only 2ry Antibodies – image taken when only secondary antibody conjugated with fluorescent dye – Goat anti-Mouse IgG Alexa488 was used.

As one can see there are no differences between left and right images, indicating no FAP staining.

A small amount of autofluorescence is visible in the green channel for 3D cultures, which does not differ in the right and left images - i.e., no FAP expression.

For each image - upper left corner - blue staining of nuclei with Hoechst dye; upper right corner - green FAP staining (Alexa Fluor 488); lower left corner, red staining of membranes with WGA Alexa Fluor 594.

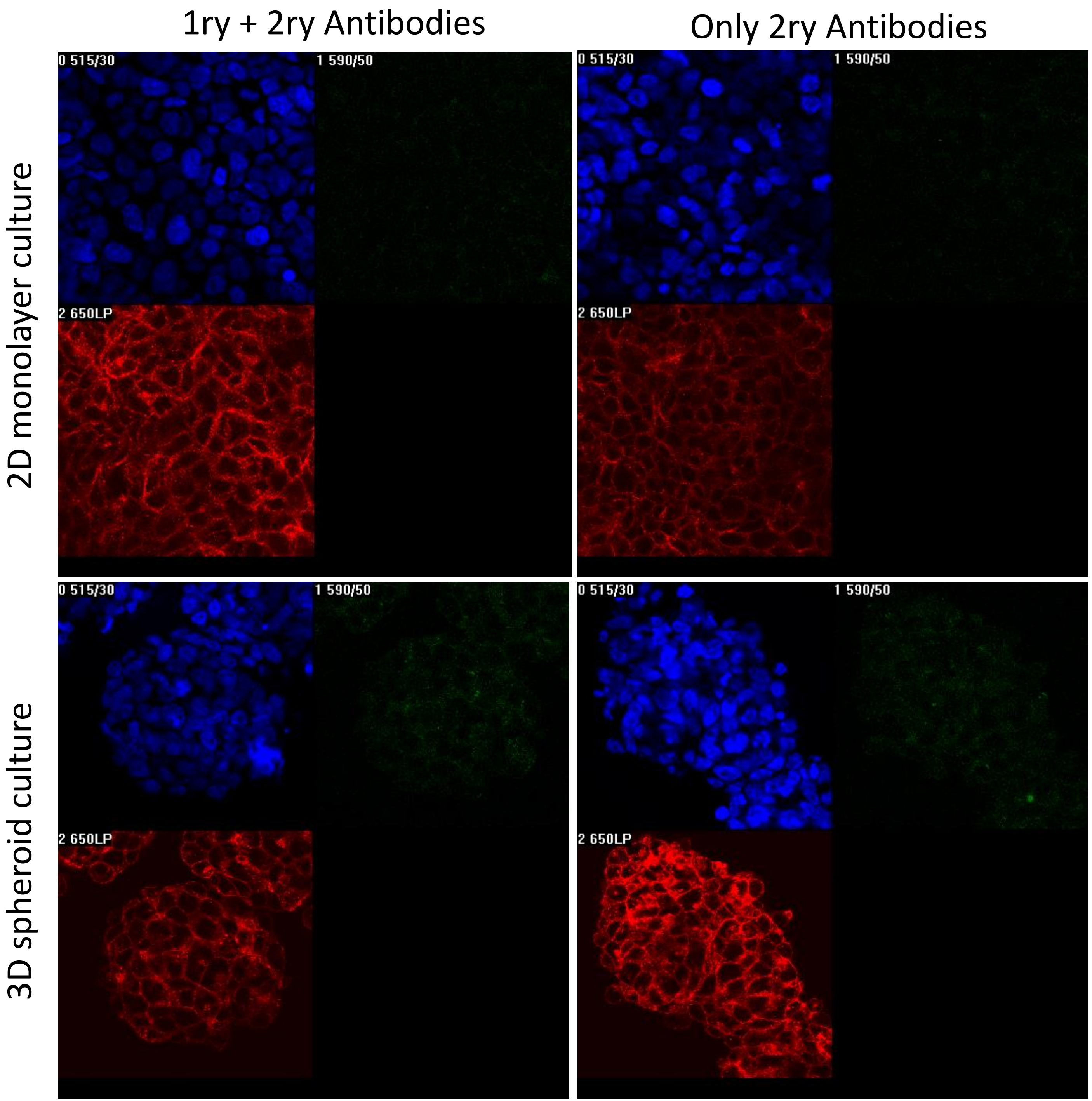

RMS FAP-positive cell line

The difference between 2D and 3D cultures of the RMS cell line is indicated.

1ry + 2ry Antibodies – image taken when primary (FAP antibody) and secondary antibody conjugated with fluorescent dye – Goat anti-Mouse IgG Alexa488 were used.

Only 2ry Antibodies – image taken when only secondary antibody conjugated with fluorescent dye – Goat anti-Mouse IgG Alexa488 was used.

As one can see there are no differences between left and right images for 2D culture, indicating no FAP staining.

In 3D cultures, the left and right images are distinct, clearly indicating the presence of FAP staining (Green Channel).

For each image - upper left corner - blue staining of nuclei with Hoechst dye; upper right corner - green FAP staining (Alexa Fluor 488); lower left corner, red staining of membranes with WGA Alexa Fluor 594.

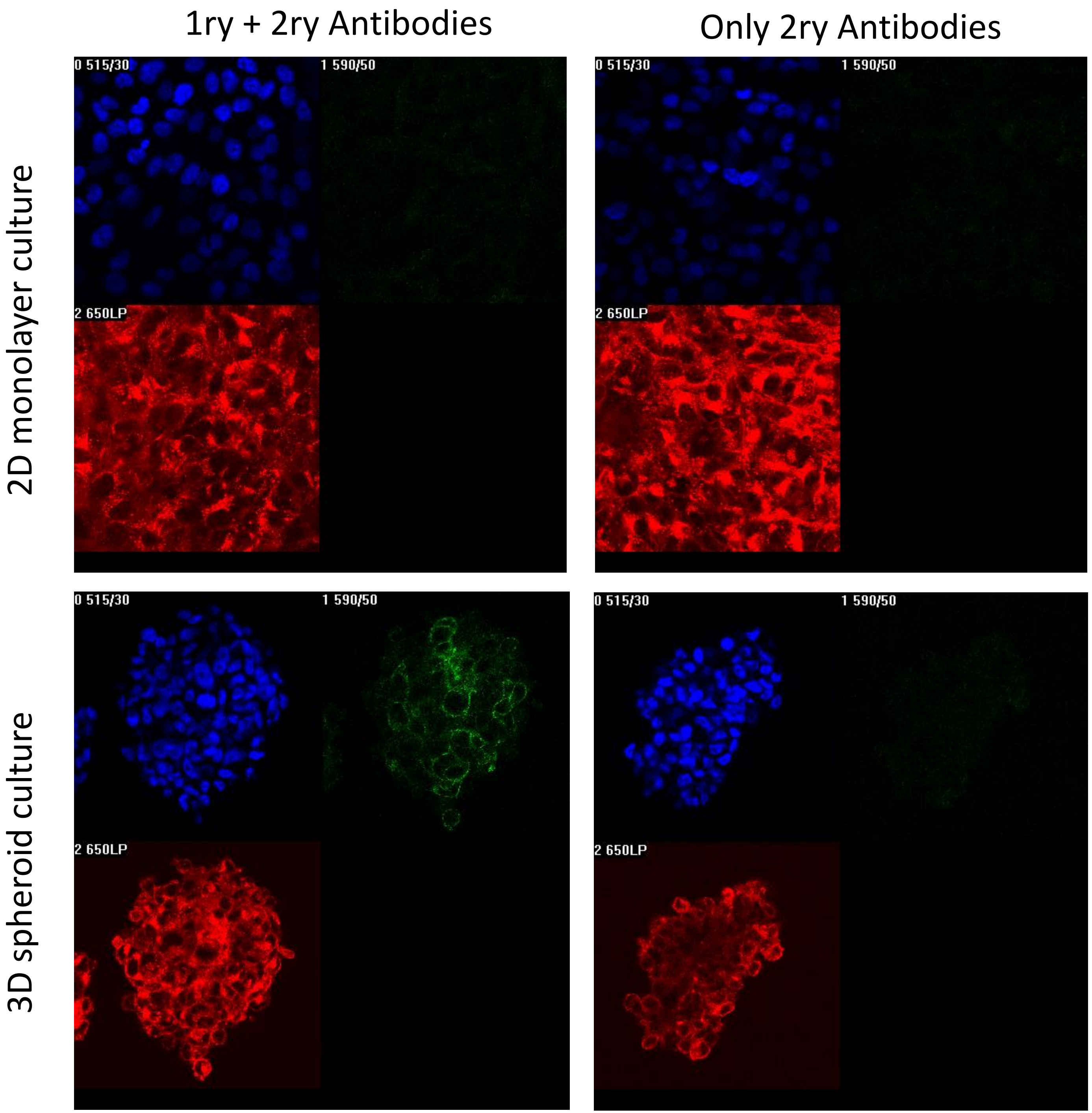

OSA FAP-positive cell line

The difference between 2D and 3D cultures of the OSA cell line is indicated.

1ry + 2ry Antibodies – image taken when primary (FAP antibody) and secondary antibody conjugated with fluorescent dye – Goat anti-Mouse IgG Alexa488 were used.

Only 2ry Antibodies – image taken when only secondary antibody conjugated with fluorescent dye – Goat anti-Mouse IgG Alexa488 was used.

As one can see there are no differences between left and right images for 2D culture, indicating no FAP staining.

In 3D cultures, the left and right images are distinct, clearly indicating the presence of FAP staining (Green Channel).

For each image - upper left corner - blue staining of nuclei with Hoechst dye; upper right corner - green FAP staining (Alexa Fluor 488); lower left corner, red staining of membranes with WGA Alexa Fluor 594.

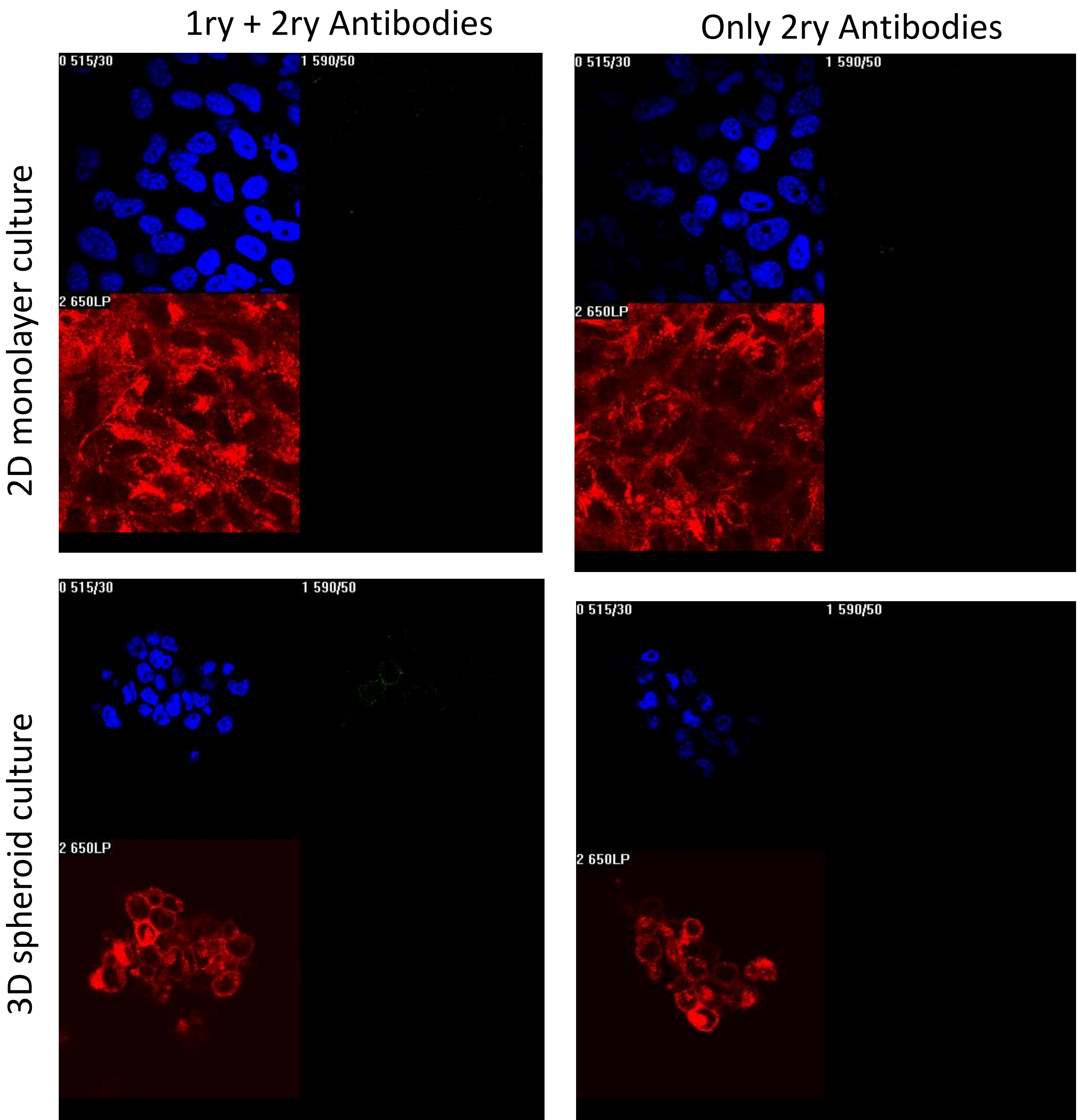

Supplement: Supplementary file 1 [file biomedicines-11-02017-s001.zip › biomedicines-2471637-supplementary.pdf]
